# The Hospital Environment as a Potential Source for *Clostridioides difficile* Transmission Based on Spore Detection Surveys Conducted at Paediatric Oncology and Gastroenterology Units

**DOI:** 10.3390/ijerph20021590

**Published:** 2023-01-15

**Authors:** Ewelina Lemiech-Mirowska, Michał Michałkiewicz, Aleksandra Sierocka, Ewelina Gaszyńska, Michał Marczak

**Affiliations:** 1 Department of Management and Logistics in Healthcare, Medical University of Lodz, 90-419 Lodz, Poland; 2 Institute of Environmental Engineering and Building Installations, Faculty of Environmental Engineering and Energy, Poznan University of Technology, 60-965 Poznan, Poland; 3 Department of Nutrition and Epidemiology, Medical University of Lodz, 90-419 Lodz, Poland

**Keywords:** *C. difficile*, C. diff Banana Broth^TM^, paediatric hospital, spores, environmental sampling, medical personnel uniform

## Abstract

*Clostridioides difficile* is an anaerobic, Gram-positive bacterium widely present in the hospital environment due to its ability to generate spores. The transfer of spores to patients through the hands of medical personnel is one of the most frequent paths of *C. difficile* transmission. In paediatric patients burdened with a serious primary illness requiring long-term hospitalisation and antibiotic therapy, *C. difficile* may be a significant risk factor for antibiotic-associated diarrhoea. The goal of the study was to assess the state of hospital environments as a potential source of *C. difficile* spores and to establish the share of hyperepidemic strains at the two paediatric units. The survey for *C. difficile* was conducted with a C. diff Banana Broth^TM^ medium, used to detect spores and to recover vegetative forms of the bacteria. Environmental samples (*n* = 86) and swabs from the clothing of medical personnel (*n* = 14) were collected at two units of a paediatric hospital, where the cases of antibiotic-associated diarrhoea with a *C. difficile* aetiology constitute a significant clinical problem. In 17 samples, a change in the broth’s colour was observed, indicating the presence of spores. Out of seven samples, *C. difficile* strains were cultured. The pathogenic isolates of *C. difficile* were obtained from swabs collected from elements of beds, a toilet, a door handle and a doctor’s uniform. In our study, we indicated points of increased risk of pathogen transmission, which could constitute a source of infection. The clothing of medical personnel may be a dangerous carrier of pathogenic spores. Periodical surveys of hospital environments with the use of specialist microbiological mediums successfully indicate the direction of corrective actions to be undertaken by the medical facility in order to increase patient safety.

## 1. Introduction

The bacterium of the *Clostridioides difficile* species is a completely anaerobic bacillus, staining positive using the Gram method. *C. difficile* is the most frequent cause of antibiotic-associated diarrhoea (AAD) hospital-associated infections. The diarrhoea occurring as a result of *C. difficile*-associated mechanisms is most frequently called *C. difficile* infection (CDI), *C. difficile*-associated diarrhoea (CDAD) or *C. difficile* colitis [1]. The main factors related to the bacteria’s virulence are the A toxin(TcdA) and the B toxin (TcdB) coded by, respectively, genes located in the PaLoc area (*pathogenicitylocus*) [2,3]. Recently, an expansion of hyperepidemic ribotypes mainly related to the BI/NAP1/027 (*North American Pulso Type 1*) clone were observed. The threat related to the hypervirulent strains results from decreased antibiotic susceptibility and from overexpression of the A toxin and the B toxin production. These strains also produce ADP-ribosyltransferase toxin, also called a binary toxin (CDT), the properties of which are similar to the toxin produced by *C. perfringens* [4,5]. In addition to the production of toxins, a very important characteristic of *C. difficile* strains is their ability to produce spores, which play a significant role in the transmission of infections. Most vegetative forms die in the low pH environment of stomach acid. Spores are resistant to conditions present in the stomach, and, in the small intestine, transform into vegetative forms able to produce the virulence factors [6]. Patients with diarrhoea caused by *C. difficile* excrete from 1 × 10^4^ to 1 × 10^7^ of these bacteria per 1 g of faeces [7]. The *C. difficile* spores may survive many weeks in the hospital environment on surfaces which are difficult to access, such as instrumentation, floors, radiators, door handles, elements of equipment inpatient rooms and toilets. The symptom-free carriers and infected patients are also a potential reservoir of *C. difficile*. The hands of medical personnel are one of the main vectors of *C. difficile* spore transmission in the hospital environment. The basic hospital procedures related to restricting the spread of *C. difficile* include, as always, following procedures related to appropriate hand hygiene, changing single-use vinyl gloves after every use, strict principles of regular cleaning and disinfection of hospital surfaces with agents with a proven sporicidal activity (sodium hypochlorite, glutaraldehyde) [8,9,10]. CDI infections were previously associated with patients over 65 years of age; however, for a long time, an increasing number of cases has been noted in other age groups, including in paediatric patients. It is estimated that *C. difficile* colonisation occurs in approximately 15–70% of infants and in approximately 5% of adults. Diarrhoea develops in approximately 25–30% of colonised patients [11]. In 2020, incidence in children and teenagers (up to 19 years of age) amounted to 307 cases in Poland (notifications delivered by medical facilities to sanitary and epidemiological stations) [12]. It is difficult to assess the actual scale of the problem, due to inaccuracies and errors related to the reporting of infections and the period of the COVID-19 pandemic, which restricted the number of planned hospitalisations. Every year, the number of *C. difficile* outbreaks drastically increases, as demonstrated by the data from the National Health Inspectorate. In 2021, the number of all *C. difficile*-related outbreaks in Poland amounted to 538, which, compared to 2020 (a total of 220 outbreaks), was an increase of144.55% [13]. A constant challenge related to the diagnostics of paediatric CDI is the lack of clear consensus regarding the definition of the disease and problems related to the indicated high level of colonisation. The rising age of a child is a significant predisposing factor for full-blown CDI, indicating the role of chronic illness as an important factor. It has been demonstrated that cancers and intestinal inflammation are related to a higher risk of occurrence of CDI in children [14].

Considering the national dynamics of proliferation of the *C. difficile* strain, the goal of our study was a microbiological analysis of the hospital environment of two paediatric units for the presence of the spores of this bacterium. We saw fit to indicate critical locations, which may significantly determine the pathogen’s transmission while influencing the CDI-related epidemiological situation in the hospital. The detection of *C. difficile* strains and their microbiological examinations were intended to establish whether the hyperepidemic strains were an important variant present in the inanimate environment of the children’s hospital. In reference to toxigenic strains, we also wanted to assess whether these were pathogens which were sensitive to standard antibiotics used in clinical practice, that is, metronidazole and vancomycin.

## 2. Materials and Methods

### 2.1. Background

Empirically, based on hospital epidemiology reports, two hospital units were selected for the study with the highest occurrence of *C. difficile*: the Pediatric Oncology Unit and Paediatric Gastroenterology Unit. At these two tertiary referral units, in 2020, a total of 27 CDI cases were detected, and 12 of them were classified as hospital-associate dinfection. Table 1 presents more detailed information from selected units reported in 2020.

### 2.2. Settings

For the prospective study material, 100 environmental samples were collected from the Paediatric Gastroenterology Unit and Paediatric Oncology Unit at a tertiary hospital in Poland;54 and 46, respectively. The samples were collected at the same time during a routine work day, without prior notification of the staff about the planned procedure (12 May 2022).

From both paediatric units, a total of 86 environmental swabs (patient rooms, bathroom, recreation room, nurse staff room) and 14 swabs from personnel’s uniforms were collected. The exact number and location of sample collection points is presented in Table 2 and Table 3 discussing research results.

### 2.3. Measurements

The study used the C. diff Banana Broth^TM^ medium (Hardy Diagnostics, Santa Maria, US), which is based on Brucella Broth supplemented with vitamin K and hemin. The L-cystine it contains enables the growth of thiol-dependent microorganisms. The germinating *Clostridioides* spores cause the fermentation of mannitol, which changes the colour of the medium from red to yellow, a positive result. The swabs were collected with sterile flocked swabs provided by the manufacturer, wetted with sterile 0.85% saline solution, and then placed in vials with the banana broth. The medium was incubated from 3 to 14 days at 37 °C, and its colour and condition were examined every 24h. Positive samples were screened to CLO and CDIFF agar media (BioMérieux, France) and to Columbia Blood Agar medium (Graso Biotech, Poland). The agar media were incubated for 48h at a temperature of 37 °C in anaerobic conditions using anaerobic culture sets—GENbag Atmosphere Generators (BioMérieux, France). Anaerobic conditions were assessed using anaerobic indicator strips (BioMérieux, France). In order to identify the *Clostridioides* strains, a Vitek 2 Compact device (BioMérieux, France) was used. In the case of detection of *Clostridioides* strains from the *C. difficile* species, genetic testing was performed using a GeneXpert device (Cepheid GmbH, Germany). XpertC.difficile BT sets were used for this purpose, which detect a sequence of genes responsible for the production of the B toxin (*tcdB*), the binary toxin (*cdtA*) and the *tcdC* base pair deletion at position 117 related to the hyperepidemic 027 ribotype strain. For toxigenic strains of *C. difficile*, their susceptibility to antibiotics used in clinical practice, that is, metronidazole and vancomycin, was tested with the use of E-tests (BioMérieux, France). These tests enabled the establishment of the antibiotics’ MIC (Minimum Inhibitory Concentration) value. The susceptibility testing was conducted based on the current version of the EUCAST guidelines (version 12.0, valid as of 01.01.2022).

## 3. Results

From the samples collected at both paediatric units, a total of seventeen positive results for the presence of *Clostridioides* spores were obtained, from which the *C. difficile* species was isolated from seven broths, and, from the remaining ten samples, other species were isolated The detailed results are presented in Table 2 for the Gastroenterology Unit and Table 3 for the Oncology Unit.

Due to the properties of the medium, aerobic bacteria did not proliferate, which was confirmed by an aerobic inspection (culturing of material on Columbia Agar medium and incubation under aerobic condition at a temperature of 37 °C for 24h). Toxigenic strains of *C. difficile* were isolated from five samples, out of which only one produced the binary toxin and was related to the ribotype 027 (generally accessible bathroom on the gastroenterology unit’s corridor). Two non-toxigenic strains of *C. difficile* were cultured (samples collected from the gastroenterology unit rooms—first from elements of the bed, second from a patient’s table). In the case of swabs collected from the clothing of personnel, a change of colour occurred in six broths, signalling the presence of *Clostridioides* spores, with *C. difficile* bacteria cultured from only one sample (uniform of a doctor from the paediatric oncology unit, a B-toxin-generating strain). Moreover, a *C. perfringens* strain was cultured from one sample (uniform of a carer, gastroenterology unit). In the remaining samples, the presence of spores related to other environmental species of *Clostridioides* was detected. In samples collected in the recreation rooms, three isolates were obtained: *C. perfringens*, *C. baratii* and *C. sporogenes*. The aforementioned microorganisms may cause infections in the patient sunder suitable conditions. In case of the recreation room, no *C. difficile* was cultured from any of the swabs.

The susceptibility of *C. difficile* strains solely producing the B toxin (*n* = 4) to metronidazole (MTZ)-assessed pursuant to MIC did not exceed 0.5 in any case, whereas, for vancomycin (VA), it was 0.25. The hyperepidemic strain demonstrated higher MIC values, which amounted to 1.0 for metronidazole and 0.75 for vancomycin, respectively. Table 4 presents averaged results of drug susceptibility of toxigenic strains of *C. difficile*.

Therefore, all strains demonstrated susceptibility to antibiotics commonly used in the treatment of CDI.

## 4. Discussion

*C. difficile* infections in paediatric patients are a very complex problem for correctly associating the microbiological result with the patient’s clinical outcome. Due to the high level of bacterium asymptomatic carrier status observed in infants and children up to 3 years of age, routine diagnostics for *C. difficile* are not recommended in this age group without precisely excluding all other infectious and non-infectious factors that can cause diarrhoeas. Nevertheless, it should be kept in mind that asymptomatic carriers in units where patients with heavy health burdens are present constitute a significant epidemiological risk, since they may participate in pathogen transmission, and thus, provide a source of contamination in the hospital environment [15,16].

In our study, the critical points related to the presence of *C. difficile* spores in the inanimate environment were: isolation rooms at the oncology unit (zones directly surrounding the patient), a general patients’ room at the gastroenterology unit (the patient’s table, in addition to elements of the bed), generally accessible bathroom (gastroenterology unit), door handles (the room located next to the patient isolation room at the oncology unit). In the case of the paediatric oncology unit, the isolation rooms were occupied by patients with a diagnosed diarrhoea with a *C. difficile* aetiology. In the isolation rooms, no locations indicating additional occurrence of spores were found outside of the bed frame. The patients did not move within the room (a child under 2 years of age stayed in their bed due to age, an older child above 5 years of age was prone due to general ill health). The younger child was present in the room with its mother (during the medical interview she was notified of the restrictions related to the child’s infectious disease, wearing a disposable single-use apron). The isolation rooms were spacious, allowing for freedom of movement during medical and care activities. In front of the isolation room, a location was set up with a sink (equipped with a soap dispenser and a hand-disinfecting liquid dispenser), adapted to the quick donning of protective clothing and gloves and to handwashing after leaving the room. Due to an active CDI infection, the direct zone around the patient obtained a positive result, which was related to the active shedding of bacteria. However, no increase in spores in any of the other nine swabs collected from isolation rooms was detected, which may result from the general principles related to the maintenance of hygiene and cleanliness, and the use of appropriate disinfecting agents. A study conducted by Davies et al. (2020) intended to establish whether contamination of the environment with CD spores during treatment with fidaxomicin was lower compared to patients treated with metronidazole or vancomycin had indicated; despite the used sporicidal agent, the spore contamination remained in about 1/3 to half of the patients undergoing treatment and after the treatment. According to researchers, this was connected to the constant production of spores and not to residual contamination, which confirms the need for thorough cleaning for the entire period the patient is present at the unit. It was demonstrated that asymptomatic carriers of *C. difficile* also have an impact on the epidemic situation of the hospital environment [17]. In our opinion, avoiding the contamination of the patient’s direct vicinity with active CDI, in particular in the case of children, is a very complex issue and complete elimination of the spores would be difficult to achieve. Important aspects concerning the limitation of the number of pathogens include issues related to the regular changing of bedsheets and their decontamination by washing using agents with proven sporicidal activity. This prevents the accumulation of spores in the direct vicinity of the patient. A study conducted by Tarant et al. (2018) indicated bed linen as an important element of hospital hygiene, which, in the case of incorrect washing procedures, may constitute a vector for *C. difficile* spore transmission [18]. Appropriate selection of detergents and high temperature reduce the risk related to the survival of the spores. At the gastroenterology unit, *C. difficile* strains were cultured from swabs collected from the elements of the bed in one of the patient’s rooms, but indicated the presence of non-toxigenic variants of the bacterium (not related to the aetiology of an infectious diarrhoea). There were three child patients with their mothers present in this room during the survey. Due to the placement of parent chairs and the small dimensions of the room, moving inside the room was difficult and caused the touching of additional surfaces. Despite the culturing of non-toxigenic strains, the presence of spores should be treated as an element that indicates a need for intervention. The asymptomatic carrying of *C. difficile* strains is not examined and identified in hospital practice, and the risk of colonisation with toxigenic strains is relatively high in a population subjected to multiple hospitalisations. In a study conducted on a group of 106 paediatric patients, colonisation with tox CD at a level of 24.5% was demonstrated. It was most frequently identified in haemato-oncology patients [19]. In our study, children present in the room in question had the ability to move, similarly to their parents, which contributes to and facilitates spore transmission. In a case when toxigenic strains would be detected, this would be connected to free transmission of the spores. Accumulation of patients present with their parents in a room with insufficient ergonomics should be thoroughly analysed.

The only hyperepidemic strain which was cultured in our study came from a generally accessible toilet intended for patients, located at the corridor of the gastroenterology unit. The toilet was used mainly by the children from the 2 closest rooms and by their parents. In a study of hospital environment Reigas et al. (2020) have presented results, in which the locations most contaminated by *C. difficile* spores were tap fixtures and toilets [20]. In case of paediatric patients, special care should be paid to the thoroughness of hand washing process, in order to prevent the transmission of spores through hands. Additionally, during the flushing of toilets bioaerosols may be created which may contribute to contamination of the room. Wilson et al. (2020) in their study have obtained 13% of positive samples before flushing the toilet and 26% after flushing, and the dominating cultured strains were the *Enterococcusfaecalis, Enterococcus faecium* and *C. difficile* [21].

The last environmental sample positive for *C. difficile* spores that we obtained was a swab from a door handle leading to a patient room located next to the isolation room at the oncology unit. Undoubtedly, hands were a factor related to the presence of *C. difficile* spores at this location. It is difficult to unequivocally establish whether the pathogen was carried in from the isolation room, or whether it came from another place or another patient, and who exactly was the spore transmission vector (medical personnel, auxiliary personnel, patient, parents spending time with the ill children). Undoubtedly, it is a location from which transmission of bacteria on hands may cascade to different surfaces, and the participation of medical personnel in this process seems the most probable scenario. The *C. difficile* spores were not detected in the room itself, which was occupied by a single patient. The daily, cyclical process of disinfection of locations in constant contact with hands (door handles, handrails, tables, switches) should be ensured.

The second area of our study was the uniforms of medical personnel. In our opinion and according to the available literature data, the clothing of medical personnel may play an important role in the transmission of *C. difficile* spores. Even a single contact by personnel with elements in the direct vicinity of the patient should be treated as presenting a high risk of contamination. An apparently clean uniform does not mean that there is not contamination with pathogenic microorganisms. During the collection of material for the study, we noticed no visible traces of soiling on the clothing of any of the employees. The swabs were collected from the vicinity of the pockets and from the front-side of the uniform (abdomen area); that is, the locations which have the most frequent contact with the user’s hands. It should be noted that although, for hands, it is possible to conduct the hygienic washing procedure every time, and thus, to break the transmission chain, the uniforms and medical clothing are used for the entire shift, and for possibly longer than one day. Single-use protective uniforms made of thin nonwoven fabric were used by employees at the unit solely during the performance of procedures on a patient in the isolation room; in the remaining cases, aprons intended for multiple uses and for periodical washing were used. As many as 35% of the isolates cultured came from hospital aprons, which indicates that this is a significant critical point related to hospital hygiene requiring control in daily practice. A positive culture of a toxigenic *C. difficile* strain was obtained from a material collected from an oncology unit doctor’s uniform. Moreover, a *C. perfringens* strain was cultured from a swab collected from the private uniform of a medical carer (gastroenterology) and other *Clostridioides* species (*C. baratii, C. innocum, C. sporogenes, C. novyi*) were cultured from the clothing of two nurses and a single employee of the cleaning team. A questionnaire survey conducted in 2021, concerning procedures related to hand hygiene and *C. difficile* transmission, with the participation of over 1600 Polish medical staff, demonstrated that during daily work, some aspects related to the area of hygiene and safety are omitted. Therefore, important elements of hospital policy related to safety and hygiene include cyclical training for personnel and controlling the performance of procedures during daily practice [22]. In earlier studies, we indicated that the hands of personnel and errors related to hand hygiene to be main factors responsible for nosocomial infections [23]. In the same manner as in the case of bed linens, high-temperature washing using recommended detergents is also an important aspect of the safe use of medical clothing. There are known practices related to circumventing hospital procedures and to washing medical clothing in locations other than the appointed ones; particularly in the case of personal clothing which is not assigned by the hospital. This carries the risk of transmission of *C. difficile* spores and of other pathogens outside of the hospital environment [24]. We did not have data regarding the frequency of uniform changes orwhat percentage of employees used the hospital laundry. The regulations in force in Poland do not exactly establish the principles for washing clothing contaminated with biological agents, including the efficiency of the used washing detergents. At the same time, these tasks may be performed by the employer or by an external company. It should be noted that, during the first wave of the COVID-19 pandemic, the healthcare system was forced to face new, unprecedented challenges. Many hours of work by medical personnel in full protective gear (FFP2/FFP3 half-mask, overalls or waterproof surgical apron, surgical cap, goggles, face shield, long and short medical gloves, protective shoes, shoe protection) became standard. Periodical shortages of personal protective equipment were common, which, in combination with the mass use of alcohol-based disinfecting agents, could have had a significant impact on the germination of spores [25]. Experiences from a hospital transformed into aCOVID-19 unit were presented by medics from the Warsaw MSWiA (Ministry of the Interior and Administration) hospital, who indicated a significant increase of CDI occurrence during the pandemic (incidence before the pandemic amounted to 2.6%, and during the pandemic it was as much as 10.9%) [26]. In the hospital featured in our study, a reduction in planned admissions and performed medical procedures occurred during the COVID-19 pandemic, which resulted in a significant decrease in the total number of all infections. In a study conducted by Kabała et al. (2022), which assessed the cleanliness of the hospital environment, four out of one hundred and sixteen collected samples demonstrated the presence of hyperepidemic strains related to the ribotype 027. A significant share of *C. perfringens* was also demonstrated, which was cultured from as many as 12 samples. The study was conducted twice: in 2017 at fourwards of a Silesian adult hospital, and in 2019 at sixwards [27]. In our study, we obtained only one isolate related to a hyperepidemic clone. Additionally, *C. perfringens* isolates were cultured from only two samples compared to the referred studies. The differences may have resulted from many aspects related to the specifics of the departments, internal hygiene and cleaning procedures, the time at which the samples were collected, the type of disinfecting agents used and the health of the patients. Referring to general epidemiology related to the *C. difficile* variants, it should be noted that, in the case of paediatric patients, the ribotype 027 is not a dominating clone and is detected in less than 20% of cases [16]. In our study, the hyperepidemic strain was cultured from the generally accessible toilet used by both paediatric patients and by their parents. It should be pointed out that transmission in this case may be multi-directional, and that the patient’s family is also at risk. The risk of occurrence of a CDI caused by a hyperepidemic strain is higher in teenagers than in younger children (it refers to patients from risk groups burdened with comorbidities) [14].

Metronidazole and vancomycin are recommended for the treatment of children in case of initial occurrence or of first recurrence of mild CDI. In 2020, fidaxomicin received FDA (Food and Drug Administration) registration, which confirmed the safety and effectiveness of use in children over 6 months of age. Administration of this drug should be considered in the case of subsequent relapses [28,29,30,31]. We established the minimum inhibiting concentrations (MIC) of the two aforementioned antibiotics, which are routinely used in the treatment of antibiotic-associated diarrhoea, on the detected toxigenic strains of *C. difficile.* All strains demonstrated susceptibility to both antibiotics. The hyperepidemic strain had significantly higher MIC values compared to standard toxigenic *C. difficile* strains. A comprehensive analysis of a large group of 215 isolates was conducted by Aptekorzet al. (2022). In the susceptibility testing of *C. difficile* isolates, susceptibility to metronidazole (GM geometric means) amounted to 0.68μg/mL (RT 027—1.0 μg/mL; RT 176—1.1 μg/mL; other toxigenic strains—0.13 μg/mL) and to 0.25 μg/mL in case of vancomycin (RT 027—0.26 μg/mL; RT 176—0.16 μg/mL; other toxigenic strains—0.22 μg/mL). Similar to our study, all strains demonstrated susceptibility to metronidazole and vancomycin. As many as 77.2% of isolates were classified as ribotype 027, from which multidrug resistance was obtained in a group of 50 isolates of RT 027 (23.3%) [32].

The C. diff Banana Broth^TM^ medium is mainly intended for the culturing and recovering of *C. difficile* spores and vegetative cells from environmental samples in accordance with the manufacturer’s assumptions. Nonetheless, it also enables the detection of other species, such as *C. perfringens*, *C. sporogenes*, *C. subterminale* and others. The listed species may be also important from the point of view of hospital epidemiology, and should form an area for further analysis.

The survey we conducted was a single-centre study. Expanding the diagnostics performed by other paediatric centres could significantly expand our knowledge on the state of the hospital environment, also possibly concerning the significance of other *Clostridioides* species. We did not have the facilities for sequencing the genetic material of the isolates. It would be recommended to cyclically repeat the surveys within a wider scope, which would enable constant monitoring of the current epidemiological situation related to the inanimate environment of the medical facility.

## 5. Conclusions

The conducted study on the state of the hospital environment confirmed the presence of *C. difficile* spores and other pathogenic species, such as, among others, *C. perfringens*. All toxigenic strains of *C. difficile* demonstrated susceptibility to metronidazole and vancomycin; however, the hyperepidemic strain obtained higher MIC values, which is an alarming phenomenon in the era of increasing drug resistance. The hyperepidemic strains did not constitute a dominating variant at the paediatric units surveyed. Taking into account the specifics of these departments, in which children with a severe course of primary disease are treated, contact with the contaminated environment may constitute a very significant risk of infection, which is why constant monitoring of the performance of procedures related to hygiene and cleanliness using media that enable the culturing of *C.difficile* is so important. The inanimate environment, which constitutes the direct vicinity of the patients and the clothes of the unit personnel in constant contact with the patients or their direct vicinity, demonstrated the presence of contamination with various species of *Clostridioides* spores. The conducted surveys indicate negligence related to maintaining cleanliness in the units and the role of medical personnel as a vector in the transmission of infections. In our assessment, actions should be undertaken to ensure higher control in the area of procedures related to hand hygiene, disinfection of rooms and also the proper use and washing of medical clothing.

## Figures and Tables

**Table 1:** Characteristics of the Paediatric Oncology Unit and Paediatric Gastroenterology Unit. Data reported in 2020.

Parameter	Oncology Unit	Gastroenterology Unit
Number of beds	57	44
Occupancy [%]	52	58
Number of hospitalisations	1826	3994
Number of discharged patients	1793	3979
Person-days	10768	9394
Bed utilisation rate	188.9	213.5
Throughput	32.0	90.8
Patient’s stay in days	6.0	2.4
Number of detected CA-CDI * cases	9	9
Number of detected HA-CDI ** cases	9	3

* CA-CDI—Community-Associated *Clostridioides difficile* infection; ** HA-CDI—Hospital-Associated *Clostridioides difficile* infection.

**Table 2:** Results of the micro biological testing conducted at the Gastroenterology Unit (*n* = 54 total samples).

	Swab Collection Location	Number of Collected Samples of a Given Type	Number of Positive Samples	Identification	B Toxin	Binary Toxin	Ribotype 027
	Patient rooms, *n* = 27						
1	Elements of the patient’s bed	6	1	*C. difficile*	no	no	No
2	Hospital chair	4	1	*C. sporogenes*	n/a	n/a	n/a
3	Light switch	4	0	*-*	-	-	-
4	Dispenser	2	0	*-*	-	-	-
5	Patient’s table	3	1	*C. difficile* *C. sporogenes*	no n/a	no n/a	no n/a
6	Window-sill/radiator	3	0	-	-	-	-
7	Oxygen switch	2	0	-	-	-	-
8	Infant weighing scale	1	0	-	-	-	-
9	Door handle	2	0	-	-	-	-
	Patient toilets, *n* = 8						
1	Door handle	2	0	-	-	-	-
2	Sink	2	1	*C. subterminale*	n/a	n/a	n/a
3	Toilet	2	1	*C. difficile*	yes	yes	Yes
4	Dispenser	2	0	-	-	-	-
	Recreation room, *n* = 8						
1	Door handle	1	0	-	-	-	-
2	Table	2	0	-	-	-	-
3	Chair	1	0	-	-	-	-
4	Computer	1	0	-	-	-	-
5	Shelves	2	1	*C. sporogenes*	n/a	n/a	n/a
6	Toys	1	0	-	-		-
	Nursing staff room, *n* = 3						
1	Table	1	0	-	-	-	-
2	Screen/computer	2	0	-	-	-	-
	Personnel’s uniforms, *n* = 8						
1	Carer	2	1	*C. perfringens*	n/a	n/a	n/a
2	Nurse	2	1	*C. baratii* *C. innocum*	n/a n/a	n/a n/a	n/a n/a
3	Physical therapist	1	0	*-*	-	-	-
4	Doctor	2	0	*-*	-	-	-
5	Cleaning team employee	1	1	*C. novyi*	n/a	n/a	n/a

n/a—not analysed.

**Table 3:** Results of the microbiological testing conducted at the Oncology Unit (*n* = 46 total samples).

	Location	Number of Collected Samples of a Given Type	Number of Positive Samples	Identification	B Toxin	Binary Toxin	Ribotype 027
	Patient rooms, *n* = 14						
1	Elements of the patient’s bed	4	0	-	-	-	-
2	Hospital chair	1	0	-	-	-	-
3	Light switch	2	0	-	-	-	-
4	Dispenser	1	0	-	-	-	-
5	Patient’s table	2	0	-	-	-	-
6	Window-sill/radiator	2	0	-	-	-	-
7	Door handle (airlock)	2	1	*C. difficile*	yes	no	No
	Patient isolation rooms, *n* = 11						
1	Elements of the bed	2	2	*C. difficile*	yes	no	No
				*C. difficile*	yes	no	No
2	Table	2	0	-	-	-	-
3	Window-sill	2	0	-	-	-	-
4	Switch	2	0	-	-	-	-
5	Door handle	2	0	-	-	-	-
6	Remote control	1	0	-	-	-	-
	Patient toilets, *n* = 7						
1	Door handle	2	0	-	-	-	-
2	Sink	1	0	-	-	-	-
3	Toilet	2	0	-	-	-	-
4	Shower tray	1	1	*C. perfringens*	n/a	n/a	n/a
5	Switch	1	0	-	-	-	-
	Recreation room, *n* = 6						
1	Table/tables	2	0	-	-	-	-
2	Chair	1	0	-	-	-	-
3	Joystick/computer	1	1	*C. perfringens*	n/a	n/a	n/a
4	Shelves	1	1	*C. baratii*	n/a	n/a	n/a
5	TV remote	1	0	-	-	-	-
	Nursing staff room, *n* = 2						
1	Table	1	0	-	-	-	-
2	Computer	1	0	-	-	-	-
	Personnel’s uniforms, *n* = 6						
1	Carer	1	0	-	-	-	-
2	Nurse	2	1	*C. sporogenes*	n/a	n/a	n/a
3	Nun	1	0	*-*	-	-	-
4	Doctor	1	1	*C. difficile*	yes	no	No
5	Cleaning team employee	1	0	-	-	-	-

n/a—not analysed.

**Table 4:** Susceptibility of toxigenic *C. difficile* strains to metronidazole and vancomycin.

Toxigenic strains	MIC MTZ	MIC VA
CD tox B(+)/isolate 1, isolation room, elements of the bed, oncology unit	0.094	0.125
CD tox B(+)/isolate 2, door handle in the air lock next to the isolation room, oncology unit	0.094	0.25
CD tox B(+)/isolate 3, doctor’s uniform, oncology unit	0.38	0.125
CD tox B(+)/isolate 4, isolation room, elements of the bed, oncology unit	0.5	0.125
CD tox B(+), tox CDT(+), 027(+)/isolate 5, toilet, gastroenterology unit	1.0	0.75
EUCAST Breakpoints	S ≤ 2	S ≤ 2

## Data Availability

The data presented in this study are available on request from the corresponding author.
